# Patient Factors Associated With Prolonged Length of Stay After Traumatic Brain Injury

**DOI:** 10.7759/cureus.59989

**Published:** 2024-05-09

**Authors:** Shameeke V Taylor, George T Loo, Lynne D Richardson, Eric Legome

**Affiliations:** 1 Department of Emergency Medicine, Icahn School of Medicine at Mount Sinai, New York, USA; 2 Department of Emergency Medicine, Mount Sinai Morningside, New York, USA

**Keywords:** traumatic brain injury (tbi), complications, prolonged length of hospital stay, major trauma, length of hospital stay (los)

## Abstract

Background

For traumatic brain injury (TBI) survivors, recovery can lead to significant time spent in the inpatient/rehabilitation settings. Hospital length of stay (LOS) after TBI is a crucial metric of resource utilization and treatment costs. Risk factors for prolonged LOS (PLOS) after TBI require further characterization.

Methodology

We conducted a retrospective analysis of patients with diagnosed TBI at an urban trauma center. PLOS was defined as the 95th percentile of the LOS of the cohort. Patients with and without PLOS were compared using clinical/injury factors. Analyses included descriptive statistics, non-parametric analyses, and multivariable logistic regression for PLOS status.

Results

The threshold for PLOS was >24 days. In the cohort of 1,343 patients, 77 had PLOS. PLOS was significantly associated with longer mean intensive care unit (ICU) stays (16.4 vs. 1.5 days), higher mean injury severity scores (18.6 vs. 13.8), lower mean Glasgow coma scale scores (11.3 vs. 13.7) and greater mean complication burden (0.7 vs. 0.1). PLOS patients were more likely to have moderate/severe TBI, Medicaid insurance, and were less likely to be discharged home. In the regression model, PLOS was associated with ICU stay, inpatient disposition, ventilator use, unplanned intubation, and inpatient alcohol withdrawal.

Conclusions

TBI patients with PLOS were more likely to have severe injuries, in-hospital complications, and Medicaid insurance. PLOS was predicted by ICU stay, intubation, alcohol withdrawal, and disposition to inpatient/post-acute care facilities. Efforts to reduce in-hospital complications and expedite discharge may reduce LOS and accompanying costs. Further validation of these results is needed from larger multicenter studies.

## Introduction

Traumatic brain injury (TBI), a condition of high burden around the world, is a leading cause of morbidity and mortality [1]. In the United States, there are over 200,000 TBI-related hospitalizations annually [2], generating 26 billion dollars in medical costs [3]. For TBI survivors, recovery from a TBI can be a long and arduous process with a significant number of days spent in the inpatient setting during the index hospitalization and post discharge in a rehabilitation facility. Longer hospital stays are associated with increasing medical costs. When compared to fatal TBI and non-hospitalized TBI survivors, TBI survivors requiring hospitalization account for approximately 80% of the total medical costs attributed to TBI [4].

Hospital length of stay (LOS) is an important outcome parameter that is directly related to healthcare utilization, medical cost, and the financial impact associated with traumatic injuries such as TBI. Across a wide range of inpatient settings, unnecessary hospital days of stay are estimated to be greater than 20% of all days that patients spend in an acute care setting [5] and are associated with poorer outcomes [6] and worse patient satisfaction with care provided by a trauma center [7]. Furthermore, previous research has found that every extra day of stay in the hospital after completing medical care was associated with 5% higher odds of complications independent of the presenting condition of the patient [8]. These findings underscore the critical need to elucidate the factors associated with prolonged length of stay to improve overall patient outcomes.

In the current literature, the definition of prolonged LOS (PLOS) varies based on the study performed. In studies of TBI patients, authors have used definitions ranging from exceeding the 75th-99th percentile for overall hospital LOS [9,10]. Outside of the TBI literature, other studies have used two standard deviations (SDs) above the mean derived cut-off [11]. This study, similar to Hwabejire et al. and their study on excessively long hospital stays after trauma [12], uses two SDs above the mean LOS as denoting an extended LOS, which for this cohort was 24 days.

We have a limited understanding of the risk factors associated with extended acute care LOS in patients with TBI. There is a dearth of literature devoted to identifying these patients and examining the factors associated with their protracted acute care stays. These TBI patients with PLOS may be a distinct subpopulation with unmet needs that create a barrier to a successful transition to discharge. A better understanding of the factors associated with PLOS in TBI patients may allow hospital systems to identify these patients earlier in the hospital course to mobilize the key resources necessary to expedite earlier disposition. Mitigation of these risk factors may lead to lower TBI-related costs, decreased complication rates, and overall better outcomes and patient and family satisfaction.

This article was previously presented as a meeting poster at the 2023 American Academy of Emergency Medicine: Mediterranean Emergency Medicine Congress on September 9, 2023, and previously posted to the Research Square pre-print server on October 23, 2023.

## Materials and methods

Data preparation

The study design was a retrospective analysis of de-identified research data that had been previously collected as part of the National Trauma Data Bank. All trauma patients who were evaluated by the trauma service at a level 2 academic trauma center and tertiary referral center between January 1, 2017, and August 30, 2022, were identified through our trauma registry. TBI was identified with Abbreviated Injury Scale (AIS) codes in the registry. Institutional informed consent was not required for this retrospective study of de-identified data based on review by our Internal Review Board.

Outcome measure

We examined extended hospital LOS. PLOS was defined as an acute hospital stay of 24 or more days from the initial trauma service evaluation in the emergency department. The LOS was calculated as the number of days between trauma service evaluation and final index hospitalization disposition. Disposition options included discharge to home or home hospice, transfer to another hospital or a long-term care setting (i.e., rehabilitation, skilled nursing facility), or death. Twenty-four days is the 95th percentile for LOS. LOS of less than 24 days were categorized as normal LOS.

Analytic and statistical approaches

The demographic and clinical characteristics of PLOS patients (LOS ≥95th percentile) were compared with normal LOS patients (LOS <95th percentile). TBI severity was classified as mild, moderate, or severe based on the Glasgow coma scale (GCS) score ranges of 13-15, 9-12, and 3-8, respectively. These GCS scores were documented on patient arrival at the emergency department. The presence of orthopedic injuries was determined based on the note of orthopedic surgeon consultation and evidence of a traumatic fracture based on the AIS diagnosis code. Ventilator use was a surrogate marker for intubation during the hospitalization. The following in-hospital complications were included as single variables: cardiac arrest, unplanned return to the operating room, unplanned intubation, unplanned extubation, inpatient alcohol withdrawal, ventilator-related pneumonia, and acute respiratory distress syndrome. A summary variable of the total number of complications for each TBI patient was calculated to denote complication burden during index acute stay hospitalization.

Descriptive statistics including means and SDs are presented for the TBI cohort of 1,343 patients. Patients with normal versus PLOS were compared using the chi-squared test or Fisher’s exact test for categorical variables and the Wilcoxon rank-sum test for non-parametric data. Statistical significance was measured at p-values <0.05. Logistic regression analysis was also performed to examine the association of PLOS with multiple predictor variables (intensive care unit (ICU) stay, GCS on hospital arrival, injury severity score (ISS), hospital discharge disposition, use of a ventilator, in-hospital cardiac arrest, alcohol withdrawal, and unplanned intubation). To assess whether collinearity affected our results, variance inflation factor (VIF), tolerance, and correlation matrices of the variables in the base regression model were evaluated for evidence of correlation. We found no evidence of an impact of potential collinearity on the results of our model. Our study employed the concordance (c) statistic (area under the receiver operating characteristic curve) and Hosmer-Lemeshow tests to assess the model’s goodness of fit. Analyses were performed using SAS On Demand for Academics (Cary, NC, USA).

## Results

We included 1,343 TBI patients in the study. The demographic and injury characteristics of this cohort are summarized in Table 1. The mean and median LOS during the index hospitalization for these patients were 7.9 days (SD = 15.7) and 4 days, respectively, with a range of 1-245 days. Of these 1,343 patients with TBI, 77 (5.7%) had hospital stays of 24 days or longer. The mean hospital LOS for normal LOS and PLOS patients were 5.3 and 50.3 days, respectively. Fall was the most frequent mechanism of injury in approximately 77% of all patients. Overall, 16% of patients had concomitant orthopedic injuries. Sample characteristics are presented in Table 2.

**Table 1:** Demographic and injury characteristics for the traumatic brain injury cohort. SD = standard deviation

Variable	Mean ± SD or n (%)
Age	66.4 ± 19.9
Sex	
Male	873 (65%)
Female	470 (35%)
Race/Ethnicity	
White	268 (20%)
Black	199 (15%)
Hispanic	338 (25%)
Asian and Pacific Islander	38 (3%)
Other	484 (36%)
Unknown	16 (1%)
Insurance	
Commercial	382 (29.1%)
Medicare	469 (35.7%)
Medicaid	295 (22.4%)
Mechanism	
Fall	1,020 (76.1%)
Motor vehicle collisions	59 (3.7%)
Intentional (violence, self-harm)	119 (8.9%)
Traumatic brain injury severity	
Mild	1,132 (84.3%)
Moderate	77 (5.7%)
Severe	134 (10%)
Glasgow coma scale score	13.6 ± 3.1
Injury severity score	14.1 ± 7.9
Orthopedic injuries	216 (16.1%)
Complications	145 (10.8%)
In-hospital death	101 (7.9%)
Length of stay	7.9 ± 15.7

**Table 2:** Sample characteristics for patients with prolonged versus normal length of stay in the traumatic brain injury cohort. *: Corresponding test statistic (chi-square, Wilcoxon) based on the analytical method. ISS = injury severity score; GCS = Glasgow coma scale; ICU = intensive care unit; TBI = traumatic brain injury; LOS = length of stay

Variable	Prolonged LOS (N = 77)	Normal LOS (N = 1266)	Test statistic*	Significance (p)
Age (mean ± SD)	65.9 ± 18.2	66.4 ± 20.1	-0.7311	0.4648
Male, n (%)	62 (80.5)	811 (64.1)	8.6411	0.0033
Length of stay (mean ± SD)	50.3 ± 44.5	5.3 ± 5.0	14.8712	<0.0001
ISS (mean ± SD)	18.6 ± 7.9	13.8 ± 7.8	5.2493	<0.0001
GCS on arrival (mean ± SD)	11.3 ± 4.2	13.7 ± 3.0	-7.1934	<0.0001
ICU days (mean ± SD)	16.4 ± 19.5	1.5 ± 3.4	10.6903	<0.0001
Ventilator days (mean ± SD)	13.1 ± 18.5	0.7 ± 2.6	12.2032	<0.0001
Alcohol present at the time of injury, n (%)	3 (14.3)	37 (9.1)	0.5884	0.4430
Race/Ethnicity				
White, n (%)	15 (19.5)	253 (20.0)	0.0115	0.9145
Black, n (%)	13 (16.9)	186 (14.7)	0.2761	0.5993
Hispanic, n (%)	22 (28.6)	316 (25.0)	0.5025	0.4784
Asian and Pacific Islander, n (%)	0 (0)	38 (3.0)	2.3785	0.1230
Other, n (%)	27 (35.0)	457 (36.0)	0.0336	0.8546
Unknown, n (%)	0 (0)	16 (1.3)	0.9849	0.3210
Insurance type				
Commercial, n (%)	21 (27.7)	361 (29.1)	0.0787	0.7791
Medicare, n (%)	22 (29.0)	448 (36.16)	1.6212	0.2029
Medicaid, n (%)	27 (35.5)	268 (21.6)	7.9463	0.0048
TBI severity				
Mild, n (%)	43 (55.8)	1,089 (86.0)	49.9069	<0.0001
Moderate, n (%)	13 (17.0)	64 (5.1)	18.7882	<0.0001
Severe, n (%)	21 (27.3)	113 (8.9)	27.2018	<0.0001
Disposition				
Home, n (%)	17 (23.9)	770 (64.2)	45.9954	<0.0001
Inpatient facility, n (%)	39 (54.9)	345 (28.8)	21.7899	<0.0001
Death during hospitalization, n (%)	15 (19.5)	86 (6.8)	16.7999	<0.0001

PLOS patients were more likely to be male (80.5% vs. 64.1%), more likely to have moderate or severe TBI (17.0% and 27.3% vs. 5.1% and 8.9%), and more likely to have Medicaid insurance (35.5% vs. 21.6%). Rates of discharge disposition differed between the two groups, as PLOS patients were more likely to be discharged to an inpatient facility (54.9% vs. 28.8%) or die during the index hospitalization (19.5% vs. 6.8%) and less likely to be discharged to home (23.9% vs. 64.2%). PLOS TBI patients also had lower GCS on arrival (11.3 ± 4.2 vs. 13.7 ± 3.0), longer ICU stays (16.4 ± 19.5 days vs. 1.5 ± 3.4 days), greater ISS (18.6 ± 7.9 vs. 13.8 ± 7.8), and more days on a ventilator (13.1 ± 18.5 days vs. 0.7 ± 2.6 days). No significant differences were observed in age, rates of alcohol present at the time of injury, rates of commercial and Medicare insurance, or racial demographics.

Significant differences in complication type and burden were observed between the two groups. Table 3 provides information on the observed complication rates and their significance. When compared to normal LOS patients, PLOS patients had higher rates of cardiac arrest (5.2% vs. 0.5%), unplanned operating room revisits (2.6% vs. 0.5%), unplanned intubation (13% vs. 1.0%), alcohol withdrawal (10.4% vs. 1.9%), ventilator-related pneumonia (5.2% vs. 0.1%), and acute respiratory distress syndrome (2.6% vs. 0.2%). When evaluating complication burden, prolonged LOS patients had a significantly higher mean number of complications per person (0.7 vs. 0.1). No significant difference was found in the rate of unplanned extubation between the two groups.

**Table 3:** Complication characteristics for prolonged versus normal length of stay patients in the traumatic brain injury cohort. *: Corresponding test statistic (chi-square, Wilcoxon) based on analytical method. Fisher’s exact test has no test statistic to report. OR = operating room; ARDS = acute respiratory distress syndrome; LOS = length of stay; SD = standard deviation

Complication	Prolonged LOS (N = 77)	Normal LOS (N = 1,266)	Test statistic*	Significance (p)
Cardiac arrest, n (%)	4 (5.2)	6 (0.5)	24.8695	<0.0001
Unplanned OR revisit, n (%)	2 (2.6)	6 (0.5)	5.4582	0.0195
Unplanned intubation, n (%)	10 (13.0)	12 (1.0)	64.6452	<0.0001
Unplanned extubation, n (%)	1 (1.3)	2 (0.2)	-	0.1637
Alcohol withdrawal, n (%)	8 (10.4)	24 (1.9)	22.2360	<0.0001
Ventilator-related pneumonia, n (%)	4 (5.19)	1 (0.1)	-	<0.0001
ARDS, n (%)	2 (2.6)	2 (0.2)	-	0.0183
In-hospital complication burden (mean ± SD)	0.7 ± 1.2	0.1 ± 0.4	6.6796	<0.0001

Logistic regression analysis for PLOS status was performed with ICU stay, GCS on hospital arrival, ISS, hospital discharge disposition, use of a ventilator, in-hospital cardiac arrest, alcohol withdrawal, and unplanned intubation as predictors. ICU stay (odds ratio (OR) = 2.5, 95% confidence interval (CI) = 1.1-5.7), disposition to inpatient facility (OR = 3.0, 95% CI = 1.6-5.9), use of a ventilator (OR = 4.1, 95% CI = 2.0-8.4), in-hospital alcohol withdrawal (OR = 3.5, 95% CI = 1.2-10.3), and unplanned intubation (OR = 3.4, 95% CI = 1.1-10.5) were significantly associated with PLOS after TBI. This model had good calibration (Hosmer-Lemeshow goodness of fit, p = 0.5606) and excellent discrimination (area under the curve = 0.840).

TBI severity was not included as a model variable for a few reasons. First, when we introduced TBI severity as a variable in the model, it was not significantly associated with LOS. Second, there were also concerns of collinearity as the VIF for GCS on arrival and TBI severity were above 10 when TBI severity was included in the model. Lastly, given the association between GCS and TBI severity, inferences could be made about TBI severity if GCS scores were significantly associated with LOS status. Ultimately, GCS on hospital arrival after TBI was not found to be associated with LOS status.

## Discussion

The purpose of this study was to provide a better understanding of the risk factors associated with PLOS in TBI patients, which is of critical importance for acute care hospitals. Reducing LOS improves the ability of the hospital to increase capacity for medical and ICU care, surgical procedures, and the transfer of patients between hospital sites [13]. PLOS contributes to increased costs, hospital overcrowding, and strained resources. Hospital overcrowding is associated with worse outcomes for the patients in these facilities [14]. Given these clinical implications and associated financial implications of increasing LOS and hospital costs, LOS has been considered a measure of the quality and efficiency of hospital processes. Prompted by agencies such as the Centers for Medicare and Medicaid, which seek to promote payments based on quality and value, demands for health systems to appropriately manage quality and efficiency measures or risk financial losses have increased [15]. As of 2015, quality metrics for payment have included LOS, with increasing LOS leading to a penalization of provider payments under the Medicare Fee Schedule [16]. Therefore, understanding the risk factors for PLOS is essential for health systems, not only to improve health outcomes but also to increase financial reimbursement for patient care.

Our study demonstrated that the TBI population at this academic trauma center was overwhelmingly male; 65% of our cohort was male. This male predominance was even greater when evaluating PLOS, with 80% of the group being male. Subgroup analysis of our cohort by sex revealed that on average males had higher ISS and lower GCS on arrival and a higher proportion of moderate or severe TBI when compared to females. Given that males had more severe injuries than females, it is reasonable that they would have a larger proportion of individuals with PLOS. Our findings are consistent with prior studies showing that males accounted for a larger proportion of TBI cohorts and had more severe traumatic injuries than females [9,10].

Our study found no significant difference in LOS as a function of age. The patients in our study were somewhat older, with an average age of 66 years, than other published TBI studies [9,10]. This older cohort reflects the mechanism of TBI as approximately 75% of our cohort had falls as the mechanism of injury, approximately 3% were caused by motor vehicle collisions, and only 9% had an injury due to an intentional or violent mechanism. This level of intentional trauma is also lower than in previous studies, where approximately 18% of injuries were found to be intentional [17]. Intentional injuries are more common in younger age groups, which may account for the differences in mechanism given the older age of our study population. The older cohort may also be a product of trauma activation criteria and hospital location at the study institution. Activations may occur retroactively when a patient is found to have intracranial hemorrhage. Older adults are more likely to have an intracranial hemorrhage in the setting of head trauma due to physiological changes in the brain and vasculature that occur with older age [18]. Other possible causes for older age in this TBI cohort are a density of older adults around the hospital (nursing homes and other residential facilities), poorer health in the older population in this area, and potential geographic modifiers (home and outdoors) that increase the risk of falls in the Harlem neighborhood where this hospital is located. Research on these topics is limited; however, one recent study in Harlem found that over 50% of older adults in their cohort had near falls in the past year [19]. Further study is needed to identify the causes of falls and subsequent TBI in this population to inform future prevention strategies.

Social determinants of health

There were significant findings in this study that highlight the importance of social determinants of health (SDoH) and acute care implications specific to this vulnerable TBI population. Healthy People 2030 defines social determinants of health as, “the conditions in the environments where people are born, live, learn, work, play, worship, and age that affect a wide range of health, functioning and quality of life outcomes and risks” [20]. Numerous recent studies have demonstrated the impact of a range of SDoH on patient health outcomes [21]. SDoH measures of interest in this study were race/ethnicity and insurance status.

There were no significant differences in racial/ethnic backgrounds between the PLOS and normal LOS groups. Results for this variable were potentially obscured by the proportion of individuals in the cohort with a racial background coded as “Other.” Almost 40% of patients in this cohort were of “Other” race. This designation may have been used for individuals who were multi-racial or had racial backgrounds that were not identified during the database inclusion process. Elucidation of the reasoning for the significant proportion of “Other” race may help identify ways to explore the racial diversity of future cohorts and determine disparities in race/ethnicity that need mitigation.

We found an association between Medicaid insurance coverage and LOS. A significantly higher proportion of PLOS patients had Medicaid insurance coverage than normal LOS patients. Interestingly, no association was found between Medicare or commercial insurance and LOS. Medicaid status has been shown to be associated with PLOS in other medical and surgical conditions [22]. Medicaid, a joint program between the federal and state governments, was created to cover the costs of providing healthcare for patients with limited financial means [23]. Adverse SDoH are prevalent in the Medicaid population [24]. These adverse SDoH are also linked to negative health outcomes [21], which makes the association between Medicaid usage and PLOS that much more important. There is a dearth of research on interventions targeting the social needs of Medicaid patients to reduce LOS after medical and surgical emergencies. In further evaluation of the association between Medicaid and LOS status, absent from our analysis is immigrant status. In New York, Emergency Medicaid often covers low-income immigrants who meet the same financial eligibility criteria as traditional Medicaid recipients but do not meet citizenship requirements [25]. Emergency Medicaid is a benefit package that includes coverage for life-threatening illnesses and hospitalizations [25]. Delays in the establishment of coverage and hospital payment in this population may play a role in increased LOS and delays in disposition. A better understanding is needed of the unique factors associated with the Medicaid population that may place these individuals at risk of PLOS to develop interventions to address patient needs, mobilize necessary resources, and reduce time to disposition.

Discharge disposition and complications

There was a higher incidence of discharge to inpatient facilities (55% vs. 29%) and death (19% vs. 7%) in the PLOS group; these patients had three times greater odds of disposition to an inpatient facility when compared to normal LOS patients. These findings are reasonable given that PLOS was associated with longer ICU stays, more severe injuries, and greater complication burden. Concomitant orthopedic injuries necessitating an orthopedic surgeon consultation appear to not have played a role in these disparities as rates of orthopedic injuries were similar between the two LOS groups. Only 24% of PLOS patients were discharged to home compared to 64% of all other patients. It is unclear based on the data whether there was a significant difference between the time the patient was deemed to have the necessary medical stability for discharge or transfer to an inpatient facility and when the actual discharge to home or facility occurred. Subgroup analysis shows that on average PLOS patients who are discharged to home spend more time in the hospital than PLOS patients who are discharged to another inpatient facility (69 days vs. 48 days), a finding that is reversed in the normal LOS population (3.6 days vs. 8.9 days). The reasons for this difference are likely multifactorial but may include concerns with insurance coverage, preparing the patient’s home for medical needs, and developing follow-up plans for new and chronic medical conditions. Further study is needed to understand the reasons for greater LOS in patients discharged to home compared to an inpatient facility in PLOS patients and if there is a delay to disposition for patients who are deemed stable from a medical standpoint.

In-hospital complications were associated with LOS. In our cohort, PLOS patients had higher rates of cardiac arrest, unplanned intubations, inpatient alcohol withdrawal, ventilator-associated pneumonia, acute respiratory distress syndrome, and a significantly greater mean complication burden than other patients. Complications, trauma-related or otherwise, have been previously shown to be associated with increased LOS [26]. These complications may serve as a barrier to medical stability and disposition as providers develop and implement treatment plans to reduce the excess burden of morbidity and mortality associated with these adverse events. PLOS patients had more severe injuries than other patients and were more likely to be intubated and have an ICU stay, leading to greater exposure to ventilators and an associated greater risk of ventilator-associated pneumonia. Importantly, regression analysis determined that unplanned intubation and inpatient alcohol withdrawal, two potentially modifiable targets for actionable intervention, predicted PLOS. The occurrence of respiratory failure necessitating an emergent intubation is a potentially preventable complication. To date, several patient-specific risk factors have been recognized as increasing the risk of unplanned intubation [27]. Identification and testing of a validated risk index may be useful to reduce unplanned intubations after trauma.

Alcohol use disorder is reported in up to 67% of trauma patients and 44% of geriatric inpatients [28]. Alcohol withdrawal in trauma patients is associated with poor outcomes [29] and highlights the need for early identification and treatment. In our cohort, the average age of individuals with alcohol withdrawal for PLOS and normal LOS were 66 years and 54 years, respectively. Geriatric trauma patients have comorbidities and concomitant diagnoses such as dementia that can complicate alcohol withdrawal assessment and treatment, whether due to chronic conditions, medications, or acute diagnoses such as TBI [30]. The development of hospital-based protocols for the early identification of trauma patients with alcohol use disorder and the management of alcohol withdrawal in trauma patients, especially older adults, may reduce the risk of subsequent PLOS and other poor outcomes in this vulnerable population.

Limitations

Our study has several limitations. This is a retrospective review of data from a single urban academic center based in a low-income largely minority area of New York City. It is possible that the results of this center may not be generalizable to TBI cohorts in other geographic regions with populations of different racial/ethnic or economic profiles. While creating the data variables for analysis in our cohort, we grouped all non-death or home dispositions into one category due to low sample distributions of multiple inpatient dispositions. Our team did not differentiate based on rehabilitation, psychiatry, or other facilities which may limit evaluation of TBI associations to specific facility types. There were also some limitations in the types of variables that we were able to ascertain for analysis based on what was captured by the registry. Variables such as the medical comorbidities and baseline health, occurrence and types of surgical procedures, time to insurance acquisition, time between medical stability and disposition, laboratory values, and vital signs were not available for analysis and could pose great value in predicting PLOS. Moreover, provider, consultant, and social work notes could have shed light on specific barriers to disposition that the registry variables failed to elucidate. The most distal time point of focus for our analysis was discharge from acute hospitalization. We did not evaluate longitudinal electronic health record data for a more comprehensive view of PLOS and distal health outcomes.

## Conclusions

In our TBI cohort based on a single urban academic trauma center, TBI patients with PLOS were more likely to have severe injuries, in-hospital complications, and Medicaid insurance. PLOS was predicted by ICU stay, intubation, alcohol withdrawal, and discharge to a post-acute care facility. The use of these predictors may facilitate early identification of TBI patients at risk of PLOS and may allow for enhanced inpatient team planning, better use of hospital resources, and the development of hospital protocols tailored to reduce complications in trauma patients. Further validation of these findings in large multicenter studies with racially diverse and nationally representative populations is warranted.
